# Association of triglyceride levels and prostate cancer: a Mendelian randomization study

**DOI:** 10.1186/s12894-022-01120-6

**Published:** 2022-10-31

**Authors:** Shusheng Zhu, Xia Hu, Yanpeng Fan

**Affiliations:** 1 Department of Urology, Jining No. 1 People’s Hospital, Jining, Shandong China; 2 Department of Geriatrics, Jining No. 1 People’s Hospital, Jining, Shandong China; 3 Department of Urology, The First Hospital of Jilin University, 71 Xinmin Road, Chaoyang District, Changchun, 130000 Jilin China

**Keywords:** Triglyceride, SNPs, Mendelian randomization, Prostate cancer

## Abstract

**Background:**

The association between triglyceride and prostate cancer (PCa) has been reported in observational studies. However, the causality from triglyceride on PCa remained unknown.

**Method:**

Two-sample Mendelian randomization (MR) was performed with triglyceride genome-wide association study (GWAS) data from 177,861 individuals and GWAS summary statistics of PCa from 463,010 individuals. Then, 48 single nucleotide polymorphisms (SNPs) of triglyceride were used as instrumental variables (IVs) to conduct MR analysis on PCa. Inverse‐variance weighted (IVW), Weighted median, MR‐Egger regression, Simple mode and Weighted mode were used for MR analysis. To verify the sensitivity of the data, heterogeneity test, pleiotropy test and leave-one-out sensitivity test were performed.

**Results:**

Association for an effect of triglyceride on PCa risk was found in IVW (odds ratio [OR]: 1.002, 95% confidence interval (CI): 1.000–1.004, *p* = 0.016). However, opposing results were observed using the weighted median (OR: 1.001, 95% CI: 0.999–1.003, *p* = 0.499) and MR‐Egger (OR: 0.999, 95% CI: 0.995–1.002, *p* = 0.401) approach. After MRPRESSO, the same result was obtained by using IVW method (OR: 1.002, 95% CI: 1.001–1.004, *p* = 0.004).

**Conclusions:**

The large MR analysis indicated that the potential causal effect of triglyceride on PCa. The odds of PCa would increase with high levels of triglyceride.

## Introduction

Prostate cancer (PCa) is the most predominant type of cancer and the second cause of death in men around the world [1]. The etiology of prostate cancer is largely unknown, and there are no identified modifiable risk factors [2, 3]. An increasing number of studies suggest a role for triglyceride in PCa development, as Rhonda et al. suggested a positive correlation [4] while Christel et al. indicated a negative correlation [5]. Scholars such as Montilla also have different views on this [6]. However, the controversy has been unsettled with regard to the true association between triglyceride and PCa.

Thus, it is necessary to disentangle the causal relationship between triglyceride and PCa. As a developing strategy for causal inference in epidemiology, MR has accomplished extraordinary victory in finding risk factors for disease. MR analysis can reduce the bias that caused by confounders or reverse causation by using the IVs to expose the causal relationship of disease-related risk factors [7]. If triglyceride has a causal effect on PCa, then variants that affect triglyceride should be expected to affect PCa proportionally. The extreme aims of this MR are to clarify the causal relationship between triglyceride and PCa.

## Methods

### MR analysis principle

**Fig. 1:**
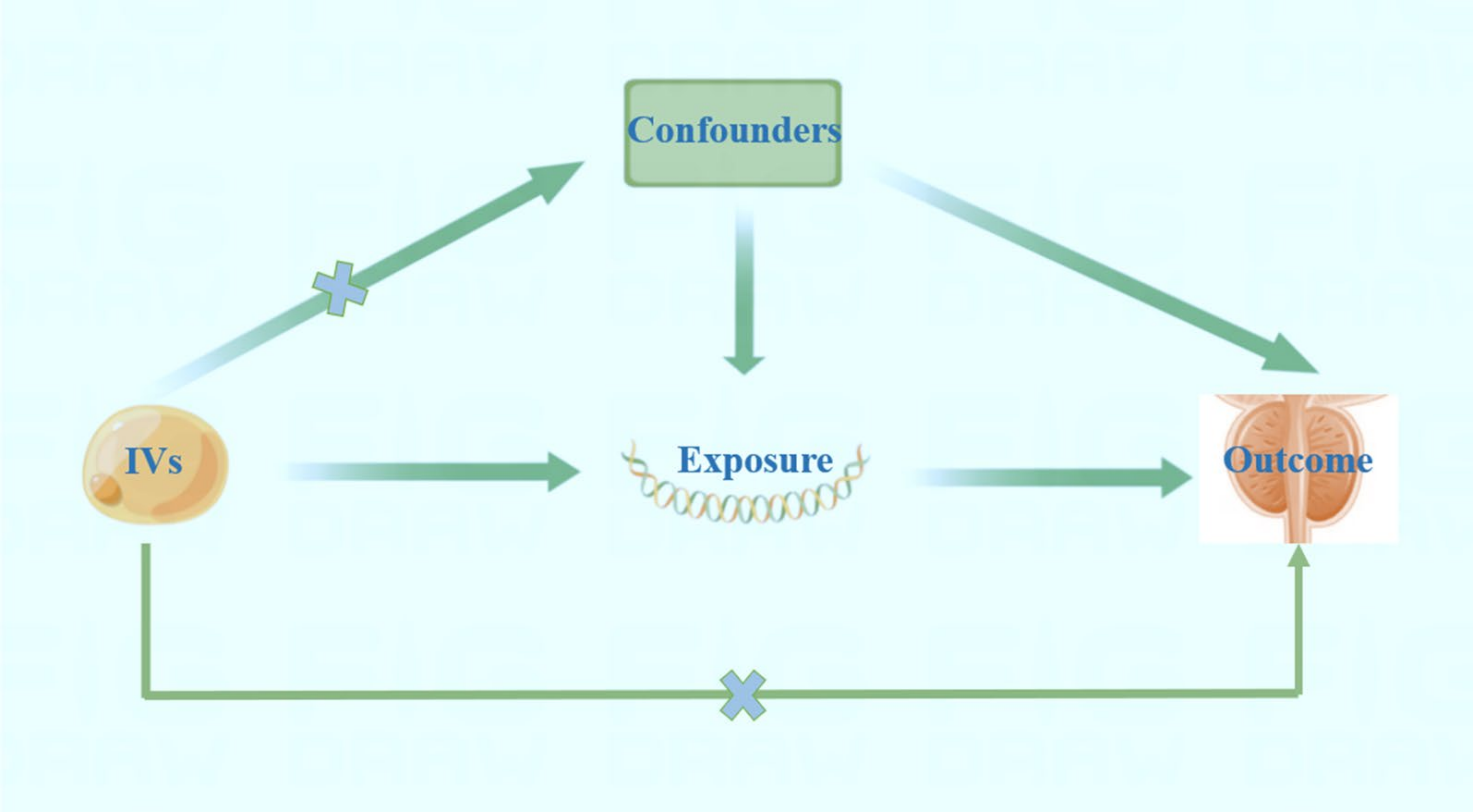
Basic assumptions of Mendelian randomization

The MR ought to be performed under three essential suspicions: (1) The genetic variants are closely linked to triglyceride; (2) the genetic variants are not linked to confounders; and (3) the genetic variants are not linked to PCa except via the way of triglyceride (Fig. 1).

### Summary statistics of triglyceride from GWAS

We extracted genetic variants of triglyceride from the open GWAS (https://gwas.mrcieu.ac.uk/,ID: ieu-a-302). The triglyceride GWAS data from 177,861 individuals, which contained 2,439,433 SNPs [8]. Then, we process the data to select appropriate IVs. 48 SNPs were significantly related with triglyceride (*p*-value < 5 × 10^–8^, linkage disequilibrium r^2^ < 0.01, clump = 1000 kb). We assessed the remaining SNPs' power utilizing the F statistics (F = beta^2^/se^2^) for each SNP and calculated a general F statistic for all SNPs. The F-statistic of 169.6 was greater than the general value of 10, indicating that genetic variants had a strong potential to predict triglyceride [9, 10].

### GWAS summary data of PCa

We used the PCa GWAS summary data from MRC Integrative Epidemiology Unit (MRC-IEU) Consortium (ID: ukb-b-2160), including 463,010 PCa individuals of European ancestry (3436 cases and 459,574 controls). A total of 9,851,867 SNPs were included in this study. The summary statistics are freely downloadable within the site. All of these data are de-identified, openly downloadable, and can be utilized without confinement.

### MR analysis and data visualization

**Fig. 2:**
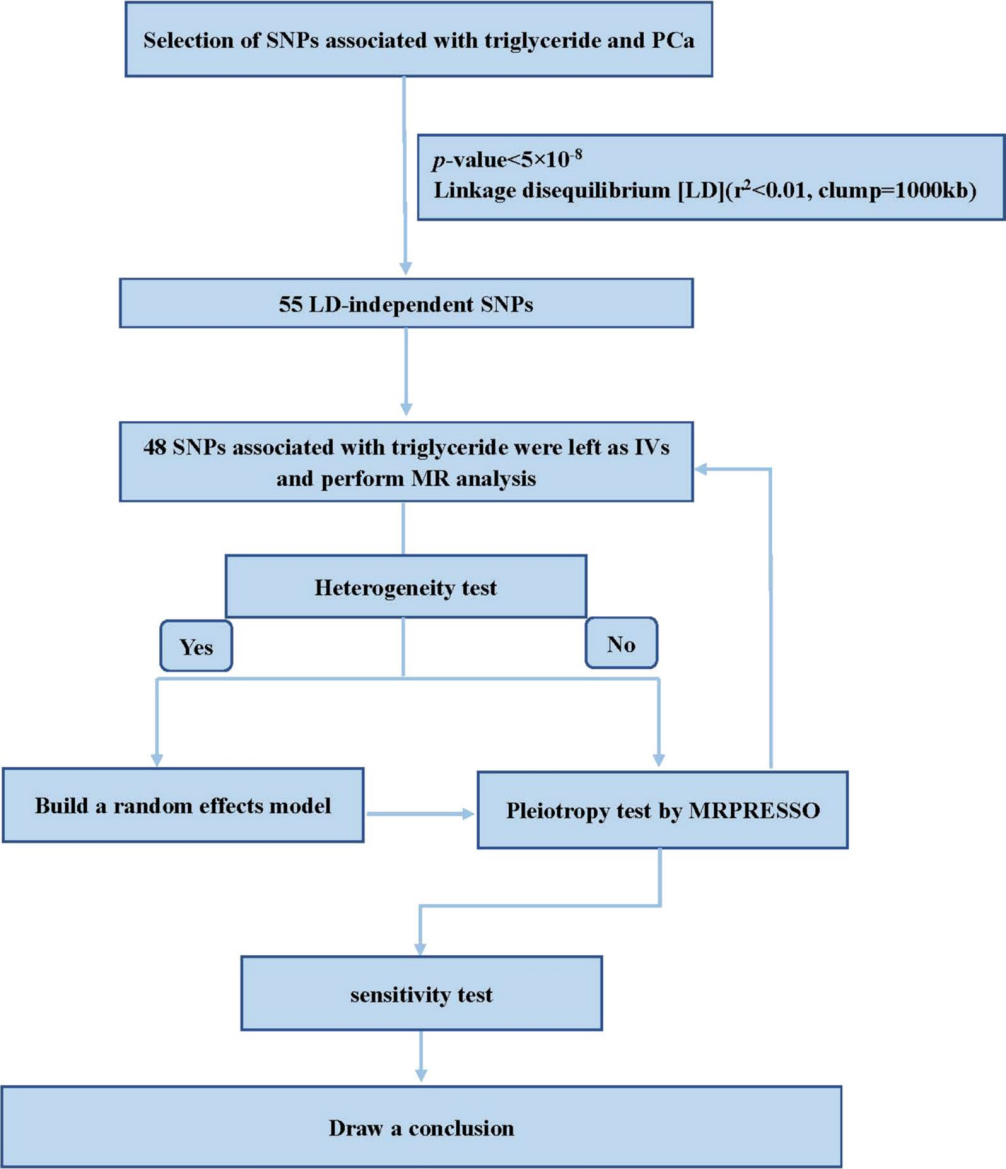
MR analysis flow chart

The design route for MR analysis can be seen in Fig. 2. The IVW method, Weighted median, MR Egger method were used to determine MR estimates of triglyceride for PCa [11–13]. Simple mode and Weighted mode as complementary methods. We used a heterogeneity marker (Cochran Q-derived *p* < 0.05) to evaluate the heterogeneity of the data [14]. MR-PRESSO [15] methods were utilized to detect horizontal pleiotropy. If the outliers were found, they would be removed and we would reassess the MR causal estimation. Details of the 48 SNPs can be found in the Table 1.

**Table 1:** 48 genome-wide significant SNPs

SNPs	Effect_allele	Other_allele	Beta	eaf	se	*p*-value
rs10440120	A	C	− 0.0306	0.1675	0.0044	5.34E − 11
rs10501321	C	T	− 0.0216	0.314	0.0035	1.41E − 08
rs10761762	C	T	− 0.027	0.467	0.0033	1.06E − 17
rs11057408	T	G	− 0.0258	0.3628	0.0035	2.05E − 12
rs11613352	T	C	− 0.028	0.1913	0.0039	9.40E − 14
rs11974409	G	A	− 0.0899	0.1939	0.0042	1.36E − 100
rs1260326	C	T	− 0.1148	0.5871	0.0034	1.00E − 200
rs12676857	C	T	0.0332	0.1544	0.0046	7.29E − 12
rs1321257	A	G	− 0.0402	0.5937	0.0034	5.99E − 31
rs13389219	T	C	− 0.0271	0.409	0.0034	2.60E − 15
rs174535	C	T	0.047	0.3628	0.0034	1.73E − 41
rs17513135	T	C	0.022	0.2322	0.0039	1.63E − 08
rs1832007	G	A	− 0.0327	0.1319	0.0047	1.72E − 12
rs2043085	C	T	− 0.0327	0.6319	0.0034	7.81E − 20
rs2068888	A	G	− 0.0241	0.4908	0.0034	1.68E − 11
rs2239520	A	G	− 0.0236	0.3734	0.0037	4.14E − 10
rs2247056	C	T	0.0378	0.7823	0.0039	3.86E − 21
rs2250802	A	G	0.023	0.6807	0.0037	1.21E − 10
rs247616	T	C	− 0.0393	0.2929	0.0037	1.12E − 25
rs2665357	C	A	0.0212	0.5092	0.0033	8.33E − 10
rs287621	C	T	− 0.0222	0.7296	0.0037	7.67E − 09
rs2954022	A	C	− 0.078	0.4697	0.0033	2.23E − 113
rs2972146	T	G	0.0281	0.6227	0.0034	2.97E − 15
rs3198697	T	C	− 0.0198	0.3826	0.0034	2.21E − 08
rs3760627	C	T	0.0189	0.4683	0.0034	5.29E − 09
rs3761445	A	G	0.0232	0.6148	0.0034	8.06E − 12
rs38855	G	A	− 0.0187	0.4736	0.0033	2.11E − 08
rs439401	C	T	0.0659	0.6201	0.0038	1.42E − 66
rs442177	T	G	0.0309	0.5528	0.0033	1.32E − 18
rs4587594	A	G	− 0.0694	0.31	0.0035	3.50E − 82
rs4719841	G	A	0.0232	0.3826	0.0034	8.86E − 11
rs4738684	G	A	− 0.0205	0.6478	0.0035	8.82E − 09
rs4810479	T	C	− 0.0474	0.7124	0.0038	2.07E − 34
rs588136	T	C	− 0.0495	0.7942	0.0041	3.37E − 30
rs634869	C	T	− 0.0272	0.562	0.0033	1.78E − 14
rs645040	T	G	0.0293	0.7691	0.004	1.83E − 12
rs676210	A	G	− 0.0733	0.2309	0.0039	3.28E − 71
rs6831256	G	A	0.0258	0.409	0.0035	1.60E − 12
rs6882076	C	T	0.0286	0.6662	0.0035	1.51E − 15
rs6995541	G	A	0.0265	0.3219	0.0037	1.34E − 12
rs719726	T	C	0.0199	0.529	0.0035	2.49E − 08
rs7248104	A	G	− 0.0222	0.4169	0.0034	5.04E − 10
rs731839	A	G	− 0.0224	0.6583	0.0036	2.65E − 09
rs749671	A	G	− 0.0211	0.3945	0.0034	6.11E − 10
rs8077889	C	A	0.0252	0.2441	0.0042	9.88E − 09
rs948690	C	T	− 0.0306	0.3047	0.0052	6.57E − 09
rs9686661	T	C	0.0379	0.1768	0.0044	2.54E − 16
rs998584	A	C	0.0293	0.5145	0.0037	3.42E − 15

The MR analysis was performed using the R packages TwoSampleMR (version 0.5.6). The MR-PRESSO was conducted using the R package MRPRESSO (version 1.0) in R program 4.1.3(https://www.r-project.org/).

## Results

### Before MRPRESSO

**Fig. 3:**
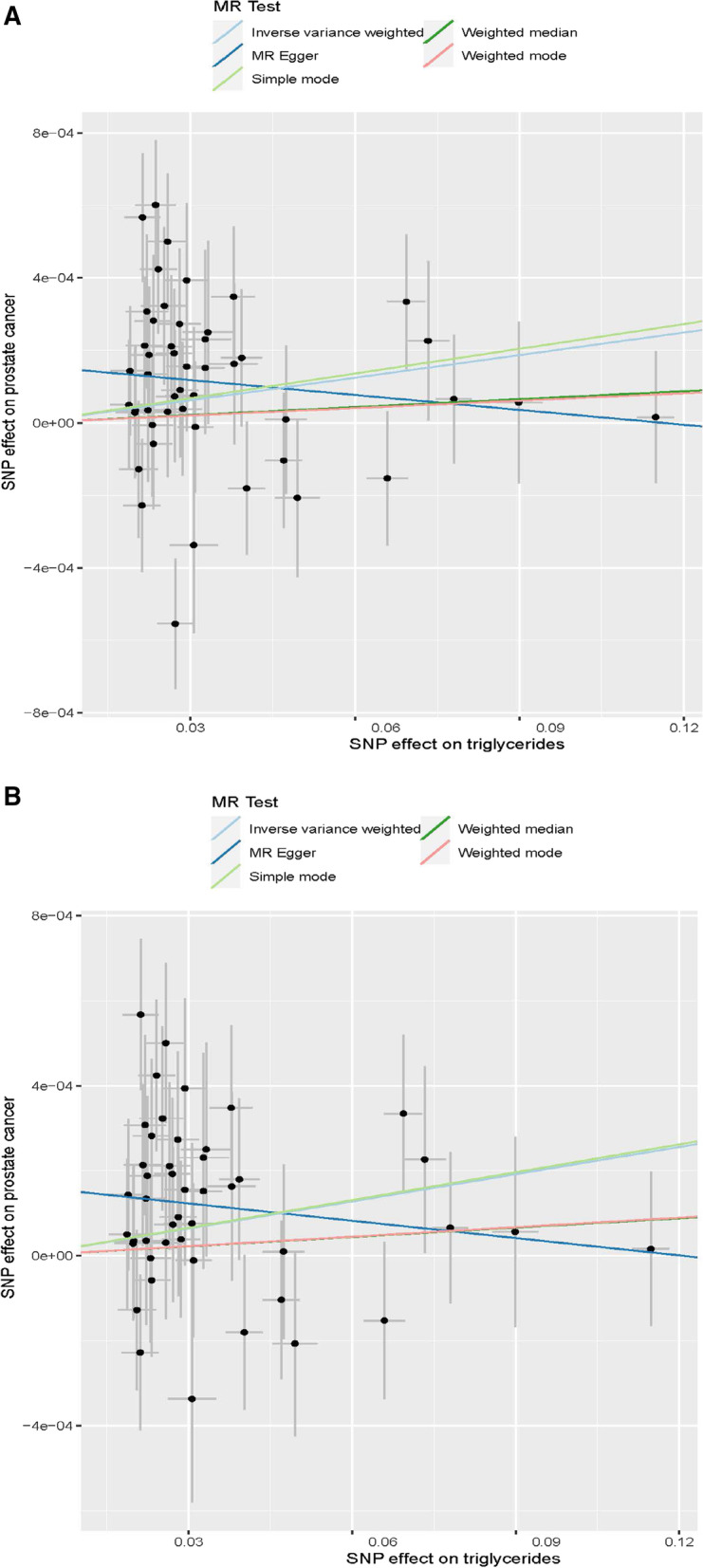
Scatter plots for MR analyses of the causal effect of triglyceride on prostate cancer. **A**: Before MRPRESSO. **B**: After MRPRESSO

**Fig. 4:**
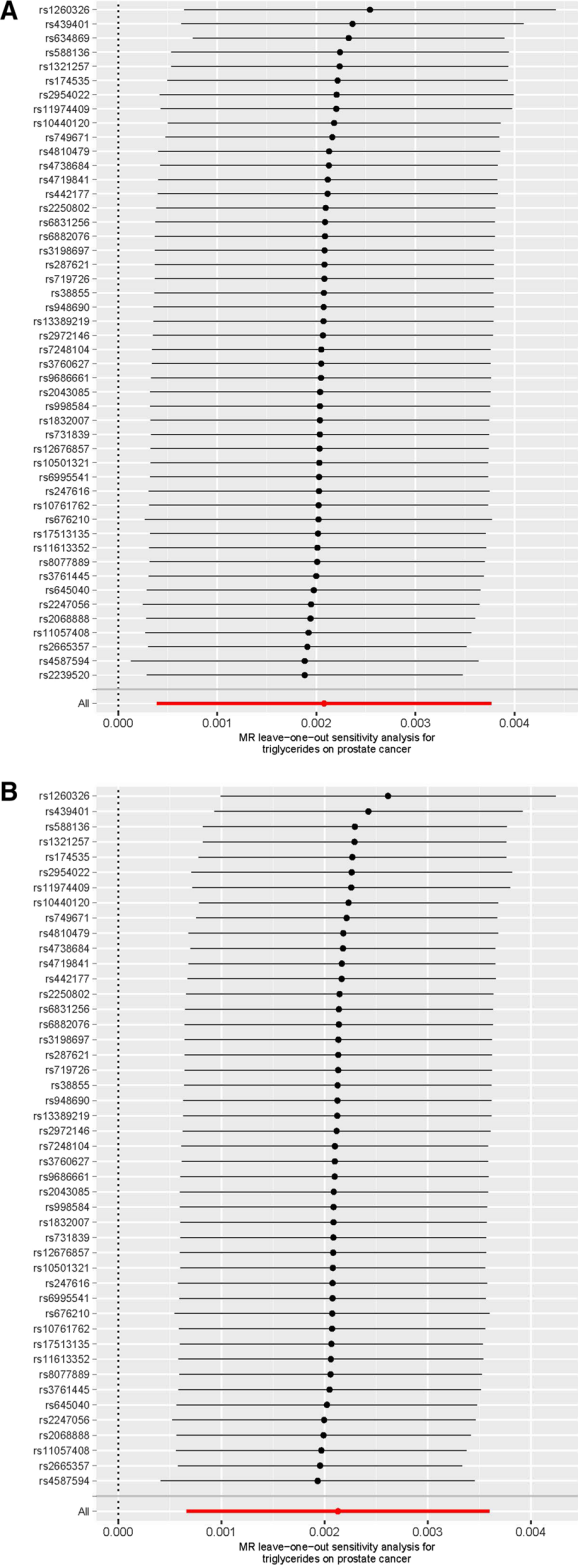
Leave-one-out of SNPs associated with triglyceride and prostate cancer. **A**: Before MRPRESSO. **B**: After MRPRESSO

**Fig. 5:**
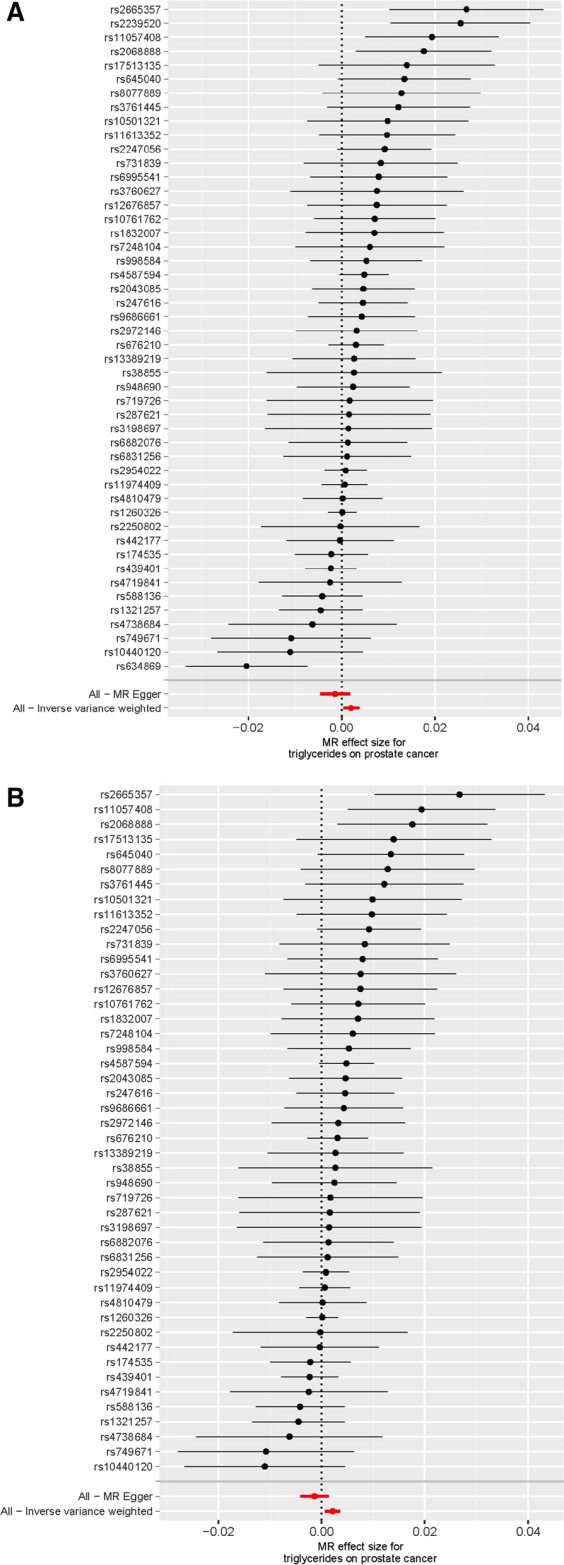
Forest plot of SNPs associated with triglyceride and prostate cancer. **A**: Before MRPRESSO. **B**: After MRPRESSO

**Fig. 6:**
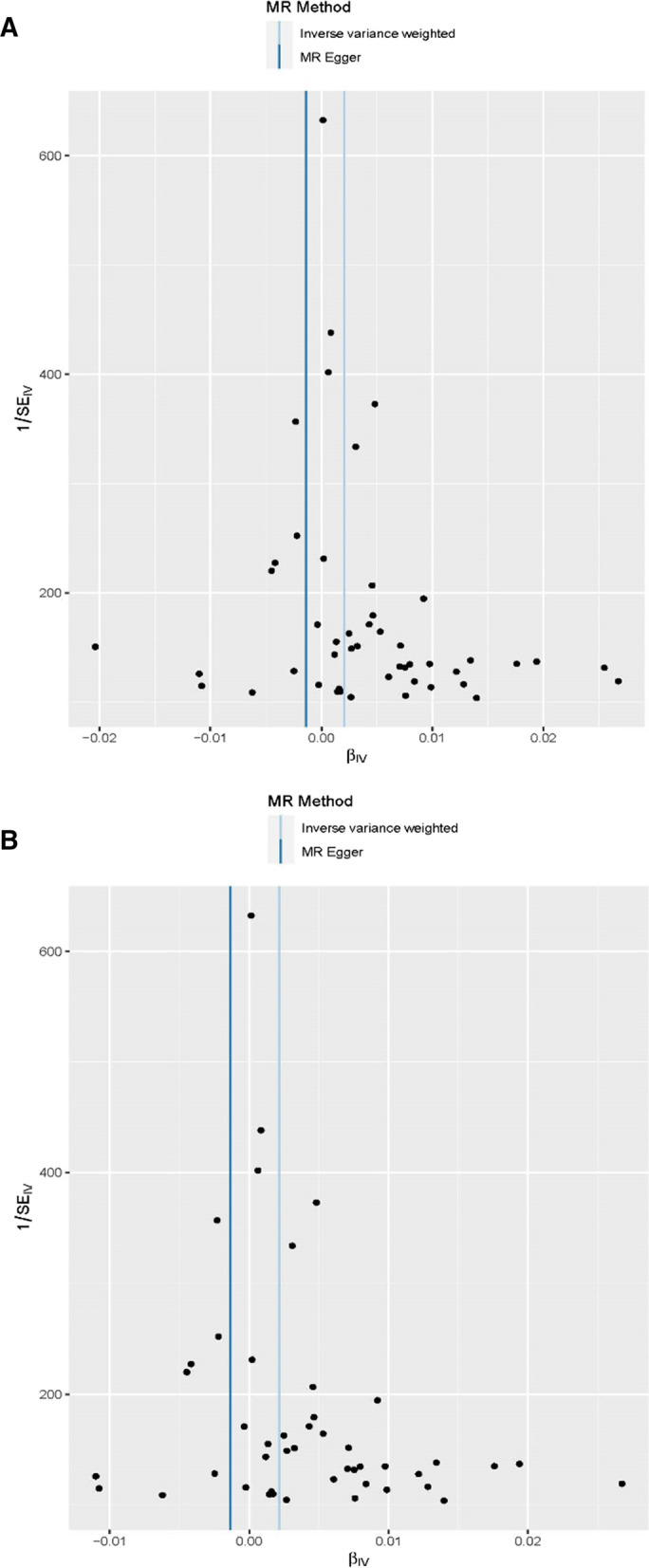
Funnel plot of SNPs associated with triglyceride and prostate cancer. **A**: Before MRPRESSO. **B**: After MRPRESSO

Utilizing the 48 triglyceride‐related SNPs, we found prove of a potential causal effect of triglyceride on the risk of PCa. In the IVW analysis, triglyceride was associated with PCa (OR: 1.002, 95% CI: 1.000–1.004, *p* = 0.016). However, different results were observed using the weighted median (OR: 1.001, 95% CI: 0.999–1.003, *p* = 0.499) and MR‐Egger (OR: 0.999, 95% CI: 0.995–1.002, *p* = 0.401) approach. We found heterogeneity in the data within the analysis of IVW (Q-value = 0.010) and MR Egger (Q-value = 0.041). Then, we directly used the IVW random effects model to estimate the MR effect size (beta = 0.002, *p* = 0.016). Horizontal pleiotropy (Global Test *P-*value = 0.011) was found by MR-PRESSO. The estimated effect sizes of the SNPs on both the triglyceride and PCa were displayed in scatter plots (Fig. 3A). No single SNP was emphatically violating the generally effect of triglyceride on PCa within the leave‐one‐out sensitivity analysis (Fig. 4A), demonstrated that MR analysis results were robust. Forest plots demonstrated that triglyceride can increase the risk of PCa (Fig. 5A). Further, the funnel plots were symmetry, demonstrating no pleiotropy (Fig. 6A).

### After MRPRESSO

**Fig. 7:**
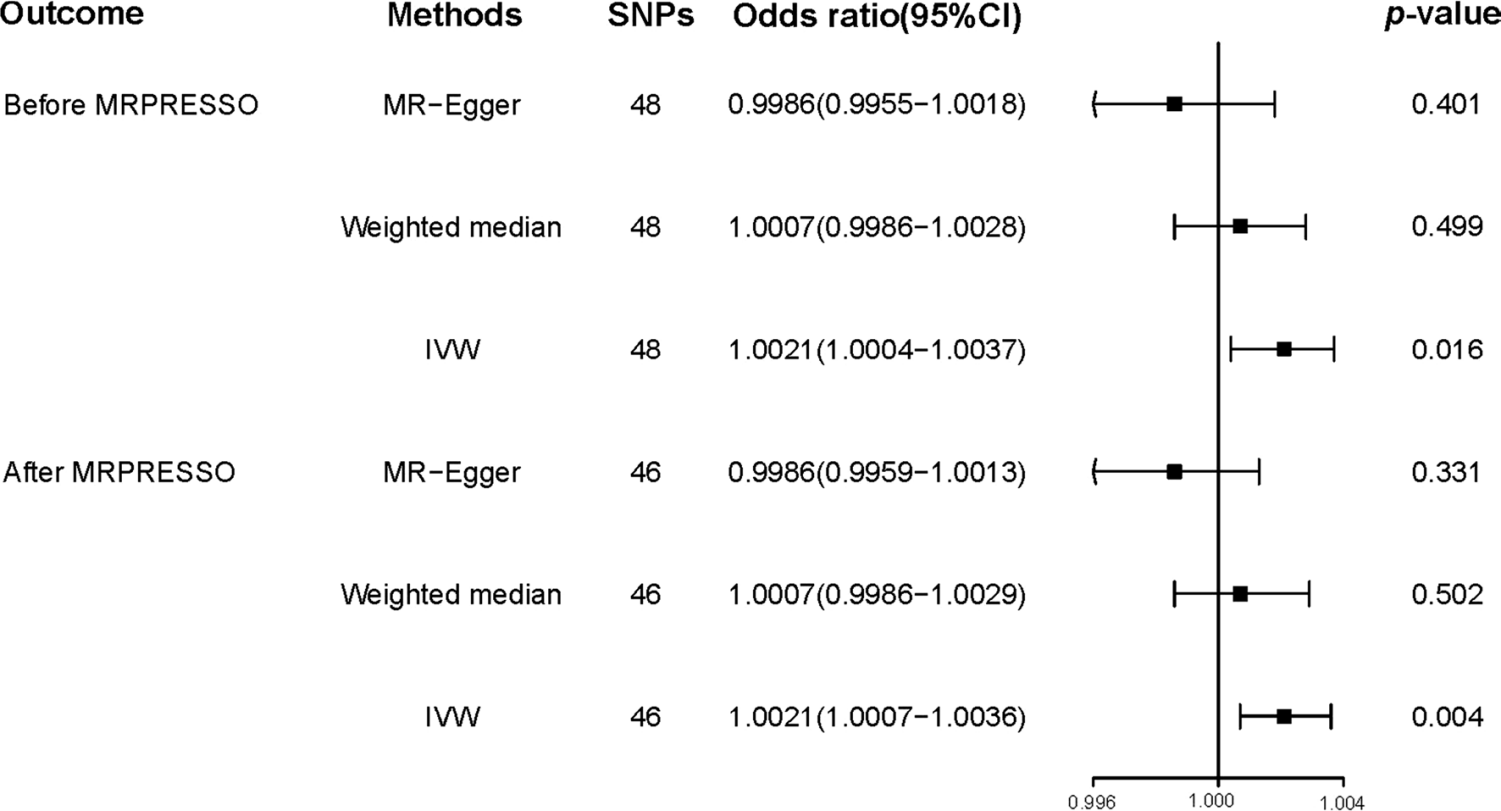
All MR analysis results

We used the MR-PRESSO package to evacuate horizontally pleiotropic IVs (rs2239520 and rs634869) with global test *p*-value < 0.05. Then, heterogeneity and horizontal pleiotropy tests were performed. No heterogeneity (IVW, Q-value = 0.235 and MR Egger, Q-value = 0.520) and horizontal pleiotropy (*p* = 0.253) were found. In the IVW analysis, triglyceride was associated with PCa (OR: 1.002, 95% CI: 1.001–1.004, *p* = 0.004). However, opposing results were observed using the weighted median (OR: 1.001, 95% CI: 0.999–1.003, *p* = 0.502) and MR‐Egger (OR: 0.999, 95% CI: 0.996–1.001, *p* = 0.331) approach. Similarly, we draw scatter plots (Fig. 3B), sensitivity plots (Fig. 4B), forest plots (Fig. 5B) and funnel plots (Fig. 6B). All MR analysis results are shown in Fig. 7.

## Discussion

We used two‐sample MR methods to comprehensively evaluate whether triglyceride levels causally influence PCa incidence and discovered potential causal effect of triglyceride on PCa.

So far, the relationship between triglyceride levels and PCa has not been elucidated [4–6]. Most previous researches were case‐control designed and failed to illuminate the causality with blurred temporal order. An inverse relationship might exist in observational studies, that elevated triglyceride levels were a consequence of PCa. Besides, observational researches could not avoid violations from confounding risk factors [16]. MR analysis was widely used to assess the causality of observed correlations, it could overcome bias by using IVs [9].

This study found that higher triglyceride levels can increase the risk of PCa. Rhonda reported that hypertriglyceridemia was positively associated with high-grade PCa [4, 17]. Evidence from experimental researches using in vivo and in vitro models showed that they may induce PCa by modulating signaling pathways, which promote carcinogenic processes such as cell growth and proliferation, oxidative stress, inflammation, and cell migration [4, 18, 19]. Triglyceride‐rich remnant like particles induce cancer by upregulating cell signaling pathways, involved in controlling cell growth and proliferation, apoptosis, and cell cycle arrest [18, 20, 21].

Our study has several major strengths. First of all, this is a MR design and suitable for causal inference. We performed a series of powerful MR methods to analyze the causal relationship between triglyceride and PCa. Second, this study consisted of MR-PRESSO parts, adding much more confidence to our research. However, our study also has limitations. The biggest concern is pleiotropy in the MR setting. Pleiotropy can be divided into vertical pleiotropy and horizontal pleiotropy. Vertical pleiotropy is hard to test, but horizontal pleiotropy can be avoided. Thus, we applied MR-PRESSO to detect the horizontal pleiotropy, hoping to minimize the bias caused by it.

## Conclusions

The large MR analysis indicated that the potential causal effect of triglyceride on PCa. The odds of PCa would increase with high levels of triglyceride.

## Data Availability

The datasets analysed during the current study are available in the open gwas repository, [https://gwas.mrcieu.ac.uk/].

## Abbreviations

PCa: Prostate cancer
MR: Mendelian randomization
GWAS: Genome wide association study
IVs: Instrumental variables
SNPs: Single nucleotide polymorphisms
IVW: Inverse variance weighted
OR: Odds ratio
95% CI: 95% Confidence interval
MRC-IEU: MRC integrative epidemiology unit
